# The Role of Mid-Regional Proadrenomedullin in the Differential Diagnosis between Culture-Negative and Culture-Positive Sepsis at Emergency Department Admission

**DOI:** 10.3390/biomedicines10020357

**Published:** 2022-02-01

**Authors:** Filippo Mearelli, Giulia Barbati, Francesca Spagnol, Alessio Nunnari, Luigi Mario Castello, Enrico Lupia, Maria Lorenza Muiesan, Salvatore Di Somma, Gian Carlo Avanzi, Gianni Biolo

**Affiliations:** 1 Unit of Internal Medicine, Department of Medical Surgical and Health Sciences, University of Trieste, Strada di Fiume 447, 34149 Trieste, Italy; francescaspagnol@yahoo.it (F.S.); alessionunnari@gmail.com (A.N.); biolo@units.it (G.B.); 2 Biostatistics Unit, Department of Medical Surgical and Health Sciences, University of Trieste, Strada di Fiume 447, 34149 Trieste, Italy; gbarbati@units.it; 3 Unit of Emergency Medicine, Department of Translational Medicine Eastern Piedmont, University of Novara, Corso Giuseppe Mazzini 18, 28100 Novara, Italy; castello@med.unipm.it (L.M.C.); avanzi@med.unipm.it (G.C.A.); 4 Unit of Emergency Medicine, Department of Medical Sciences, University of Turin, Corso Bramante 88, 10126 Turin, Italy; enrico.lupia@unito.it; 5 Unit of Internal Medicine, Department of Clinical and Experimental Sciences, University of Brescia, Piazzale Spedali Civili 1, 25123 Brescia, Italy; marialorenza.muiesan@unibs.it; 6 Unit of Emergency Medicine, Department of Medical Surgery Sciences and Translational Medicine, University “Sapienza” of Rome, Via di Grottarossa 1035/1039, 00189 Rome, Italy; salvatore.disomma@uniroma1.it

**Keywords:** sepsis, culture-negative sepsis, biomarkers, emergency department, C-reactive protein, lactate, procalcitonin, mid-regional proadrenomedullin, soluble triggering receptor expressed on myeloid cell-1, presepsin, soluble phospholipase A2 group IIA and soluble IL-2 receptor α

## Abstract

Background: The host response in culture-negative sepsis (CnS) has been marginally explored upon emergency department (ED) admission. It would be of paramount importance to create a clinical prediction rule to support the emergency department physician in identifying septic patients who can be treated with antibiotics immediately without waiting time to draw cultures if they are unlikely to provide useful diagnostic information. Methods: A multivariable logistic regression analysis was applied to identify the independent clinical variables and serum biomarkers of the culture-negative status among 773 undifferentiated septic patients. Those predictors were combined to build a nomogram predictive of CnS. Results: The serum concentrations of six biomarkers, among the eight biomarkers assayed in this study, were significantly lower in the patients with CnS (449) than in those with culture-positive sepsis (324). After correction for co-variates, only mid-regional proadrenomedullin (MR-proADM) was found to be independently correlated with culture-negative status. Absence of diabetes, hemoglobin concentrations, and respiratory source of infection were the other independent clinical variables integrated into the nomogram—its sensitivity and specificity for CnS were 0.80 and 0.79, respectively. Conclusions: Low concentrations of MR-proADM were independently associated with culture-negative sepsis. Our nomogram, based on the MR-proADM levels, did not predict culture-negative status with reasonable certainty in patients with a definitive diagnosis of sepsis at ED admission.

## 1. Introduction

According to the Surviving Sepsis Campaign guidelines, one of the first measures to improve the outcome of sepsis is the administration of intravenous antimicrobials as soon as possible and within 1 h of its recognition. Blood cultures should be obtained before starting antibiotics if doing so results in no substantial delay in their administration [1]. Antimicrobials are increasingly used in the pre-hospital setting [2]; thus, at emergency department (ED) admission, blood cultures are frequently obtained during concurrent antibacterial therapy. Moreover, in clinical practice, if blood cultures are difficult to draw (e.g., shock), priority is given to antibiotics. On the one hand, such an aggressive approach has improved the outcome of sepsis [1], but, on the other hand, it has rendered microbiological diagnosis even more challenging since blood culture yield seems to be affected by concomitant antimicrobial administration [3,4,5].

Blood culture-negative status is becoming increasingly common [6]. Many factors other than concurrent or previous antibacterial therapy have been implicated in its genesis, such as sepsis mimickers; infections caused by fastidiously growing bacteria; and non-culturable bacteria, viruses, and fungi [2,6,7,8,9,10,11,12,13,14,15,16,17]. The understanding of culture-negative sepsis (CnS) becomes even more complex when the definition of negative cultures—historically, the byword for blood culture negativity in the sepsis literature—is extended to all cultures. In fact, based on the assumption that the rate of sampling of any biological sample other than blood cannot be deduced in nearly all the studies conducted in this field [2,6,7,8,9,10,11,12,13,14,16,17], it becomes impossible to exclude that CnS could be the result of insufficient work with respect to culture-positive sepsis (CpS). Finally, given the above limitations, the inflammatory status of the host has only been retrospectively and marginally explored in the two groups [2,10,12,13,15]. Therefore, in this secondary analysis of a multicenter prospective cohort study involving 1132 adult patients with suspected sepsis in the ED, we aimed to:

-establish whether culture-negative status is characterized by unique features of the host response

-evaluate whether a new clinical prediction rule could properly identify CnS among patients definitively diagnosed with sepsis.

## 2. Methods

**Figure 1:**
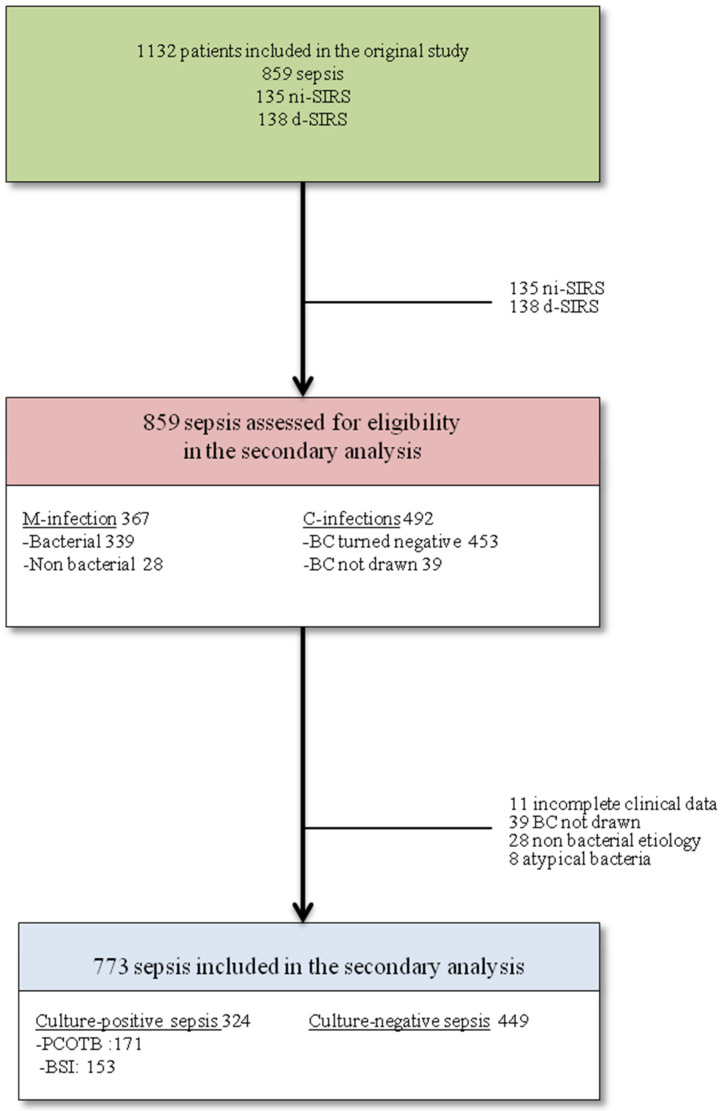
Flow chart of enrollment in this study. List of abbreviations: non infective SIRS = ni-SIRS, d-SIRS = debatable SIRS, M-infections = microbiologically documented infections (definition available in the Supplementary Materials), C-infections = clinically documented infections (definition available in the Supplementary Materials), BC=blood cultures, PCOTB = positive cultures other than blood, and BSI = bloodstream infections.

A secondary analysis was performed using data from the “Need Speed Study” database [18], which was a multicenter prospective study that aimed to derive and validate a predictive algorithm that could robustly differentiate sepsis from non-infectious systemic inflammatory response syndrome (ni-SIRS) at ED admission. The original study was conducted from 15 March 2013 to 15 March 2015. A total of 1132 patients with suspected sepsis fulfilling the Sepsis-2 criteria were enrolled in 5 Italian EDs. In brief, at the end of clinical work-up or death, patients were categorized as having sepsis, ni-SIRS, or debatable SIRS (d-SIRS). The source, etiology, and severity of sepsis were adjudicated according to a pre-defined classification system (see Supplementary Materials). The following cohorts were excluded from this sub-analysis: ni-SIRS, d-SIRS, septic patients with incomplete clinical data, septic patients in whom at least two sets of blood cultures were not drawn at ED admission, patients with a diagnosis of non-bacterial sepsis, and patients with a diagnosis of sepsis due to atypical bacteria originating from the lower respiratory tract (Figure 1).

### 2.1. Definitions

CpS was defined as sepsis in which one or more viable bacteria were cultured in blood (BSI) or in other biological specimens of the suspected source of infection (i.e., positive cultures other than blood [PCOTB]). Blood culture-negative sepsis included CnS and sepsis with PCOTB (i.e., all septic patients with negative blood cultures). CnS was defined as sepsis in whom bacterial cultures were negative in both the blood and biological specimens of the suspected source of infection. Comorbidities and organ dysfunctions were defined as reported in the Supplementary Materials.

### 2.2. Biomarkers Assay

At ED admission, the serum concentrations of C-reactive protein, procalcitonin, lactate, soluble phospholipase A_2_ group IIA (sPLA_2_GIIA), presepsin, soluble IL-2 receptor α (sIL2Rα), and soluble triggering receptor expressed on myeloid cell-1(sTREM-1) were assayed as previously reported [18]. Mid-regional proadrenomedullin (MR-proADM) was measured by an automated Kryptor analyzer (Kriptor Brahms AG, Hennigsdorg Germany).

### 2.3. Outcomes

The primary outcome of the study was to identify the independent biomarkers of CnS and to relate their serum concentrations with the reasons for culture-negative status. Secondary outcomes were to derive a nomogram predictive of CnS and to evaluate its performance in identifying culture-negative status among patients definitively diagnosed with sepsis at ED admission.

### 2.4. Statistical Analysis

For descriptive statistics, categorical data were summarized as absolute frequencies and percentages, and continuous data were summarized as medians with interquartile ranges. A Pearson’s chi-square test was used to analyze the categorical variables and the Mann–Whitney test was used to analyze the continuous variables. The variables were further examined for association with CnS by univariable logistic regression analysis. The unadjusted odds ratios (ORs) and their 95% confidence intervals (CIs) were calculated. Starting from a full multivariable model containing all candidate predictors with an unadjusted *p* value ≤ 0.1, a selection procedure was applied based on finding the best subset of parameters [19] and a nomogram predictive for CnS was created (the details are available in the Supplementary Materials). To specifically evaluate the additive diagnostic value of MR-proADM, the discrimination power of two nested models (with or without the biomarker) was quantified using C statistics (area under the receiver operating characteristic curve [AUROC]), and the De Long test was calculated to compare values. According to the Youden Index method, a cut-off was determined, and sensitivity, specificity, positive predictive value, and negative predictive value were calculated. Finally, the adjusted ORs of the biomarkers associated with the three cultural statuses (CnS, PCOTB, and BSI) were estimated by means of multinomial multivariable models, and the distribution of biomarkers across groups was visually represented through a violin plot. All tests were two-tailed and a *p* value less than 0.05 was considered statistically significant. All analyses were performed using IBM SPSS statistic 24 software and R software, R Core Team (2019), using the libraries “rms,” “Hmisc,” “bestglm,” “OptimalCutPoints,” and “ggplot2.”

## 3. Results

The present study included 773 patients who received a definitive diagnosis of sepsis at the end of clinical follow-up among the 859 enrolled in the original study (Figure 1). The baseline characteristics of the 449 CnS patients and 324 CpS patients are described in Table 1.

Chronic heart failure, previous acute myocardial infarction, and chronic pulmonary diseases were significantly associated with CnS (*p* = 0.016, *p* = 0.019, and *p* < 0.001, respectively). Diabetes and prosthetic devices were more frequent in CpS than in CnS (*p* = 0.006 and *p* < 0.001, respectively). If compared to patients with CpS, patients with CnS received antibacterial therapy—during their stay in ED—less frequently (6 [2%] vs. 26 [6%], *p* = 0.009). Lower respiratory tract infections (LRTIs) were more common in the patients with CnS than in the patients with CpS. The other single sources of infection, grouped as non-LRTI, were the most frequent source of infection in CpS. The median concentrations of procalcitonin, sIL2Rα, presepsin, and MR-proADM were significantly lower in patients with LRTIs than in patients with non-LRTIs (*p* < 0.001, *p* = 0.002, *p* < 0.001, and *p* = 0.008, respectively). The proportion of patients with at least one biological sample other than blood was similar between CnS and CpS patients (*p* = 0.205, Table 1). The pathogens involved in CpS are shown in Table S1. CnS and CpS were also similar in terms of their median Sequential Organ Failure Assessment (SOFA) score and mortality. However, CnS and CpS were associated with specific types of organ failure (Table 2)—the former with respiratory failure (*p* < 0.001), acute decompensated heart failure (0.002), and acute coronary syndromes (0.002), while the latter with neurologic (0.002) and renal dysfunction (0.007).

**Figure 2:**
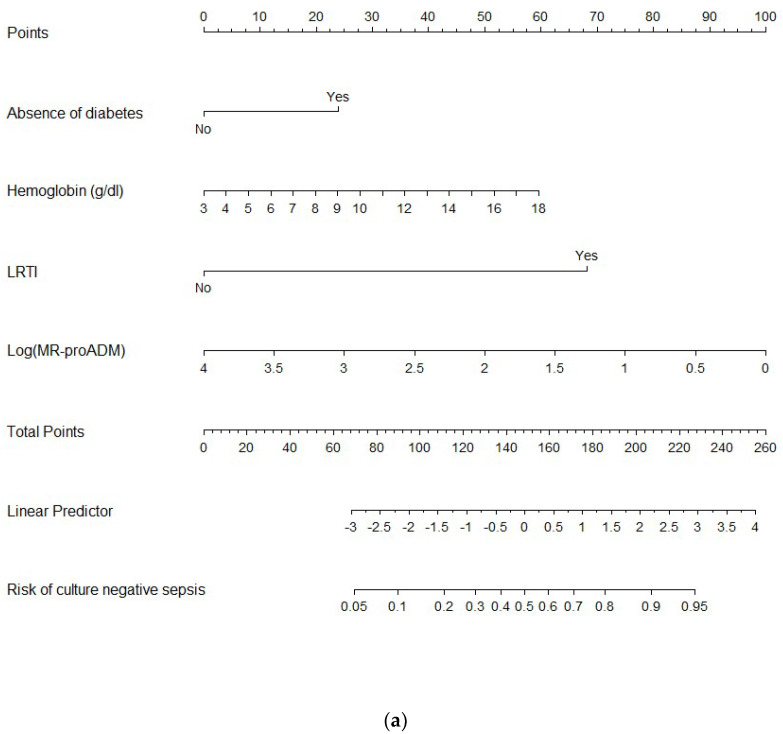

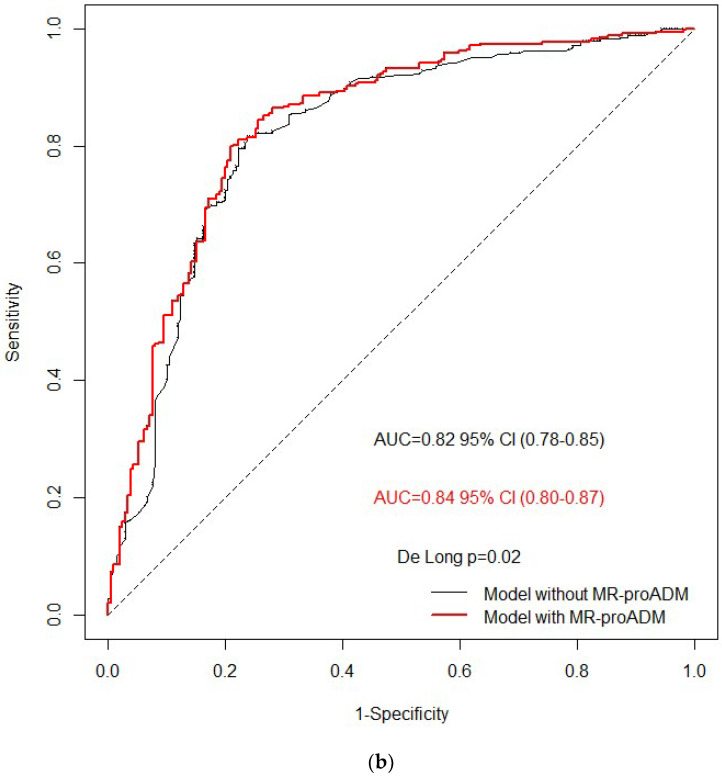
(**a**) The nomogram to predict culture-negative status among definitively diagnosed septic patients. List of abbreviations: LRTI = lower respiratory tract infection and MR-proADM = mid-regional proadrenomedullin. Its ROC curve and AUROC are available in Figure 2b. (**b**) Performance of the nomogram with or without mid-regional proadrenomedullin (MR-proADM) to predict culture-negative sepsis: receiver operating characteristic curves (ROC) and area under ROC curves (AUC).

The median concentrations of all the biomarkers, except lactate and sTREM-1, were lower in CnS than in CpS (Table 1); after correction for covariates, only MR-proADM was independently correlated with culture-negative status (see Table S2). MR-proADM and the other independent predictors of CnS (absence of diabetes, hemoglobin concentrations, and the lower respiratory tract as a source of infection) were used to build the nomogram (Figure 2a).

The sensitivity, specificity, negative predictive value (NPV), positive predictive value (PPV), negative likelihood ratio, and positive likelihood ratio of the nomogram to predict CnS (according to a cut-off of 0.59) are available in Table S3.

**Figure 3:**
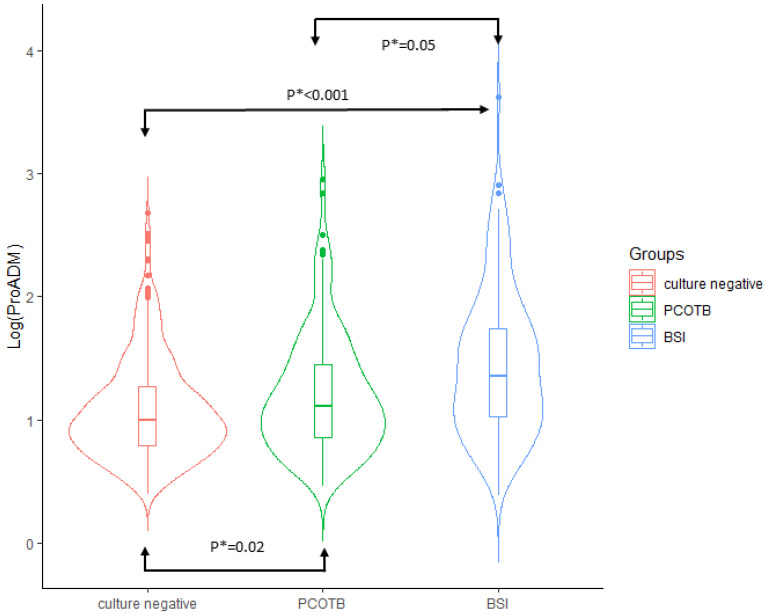
Correlation between median MR-proADM concentrations and the three cultural statuses (violin plot): culture-negative sepsis, sepsis with positive cultures other than blood (PCOTB), and bloodstream infections (BSI).

The comparison between CnS, POCTB, and BSI in terms of demographics, comorbidities, clinical variables, host response, and source and severity of infection is reported in Table S4. In the multinomial logistic regression analysis, MR-proADM was the only biomarker that was significantly different between all three groups independent of covariates (age, diabetes, prosthetic devices, and source and severity of sepsis; Figure 3).

## 4. Discussion

The first objective of this study was to identify the independent serum biomarkers of CnS at ED admission, when the results of cultures are usually not available. The levels of C-reactive protein, procalcitonin, lactate, sPLA_2_GIIA, presepsin, sIL2Rα, sTREM-1, and MR-proADM were measured at ED admission in 773 patients with definitive diagnosis of sepsis; at the end of microbiological work-up, 449 and 324 of the septic patients were deemed to be CnS and CpS, respectively. In previous studies [10,14], the analysis of the inflammatory state of the host in patients with CnS and CpS was mainly focused on C-reactive protein and procalcitonin—the serum concentrations of both biomarkers were proved to be significantly blunted in CnS [10,14]. Among the eight biomarkers assayed in our study, only MR-proADM was independently associated with culture-negative status. If compared with CpS, CnS exhibited lower serum concentrations of that biomarker. We also observed that only the levels of MR-proADM were significantly different between the three cultural statuses of sepsis highlighted in this study. The concentrations of MR-proADM increased starting from CnS (lowest levels), through POCTB (intermediate levels), and ending with BSI (highest level; Figure 3).

To our knowledge, no studies exist that have explored the correlation of the concentrations of MR-proADM with the aforementioned end points in septic patients.

**Figure 4:**
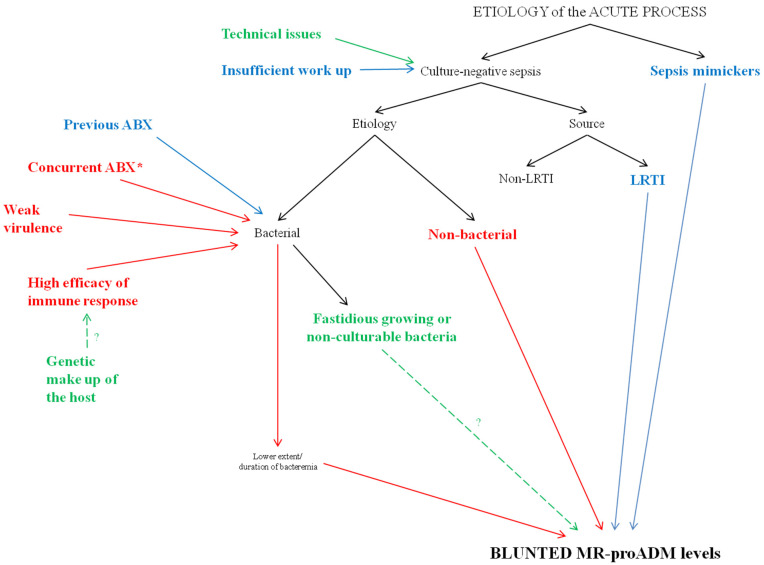
Motivations of culture-negative status: the hypothetical size of their impact on the genesis of culture-negative sepsis in this study. List of abbreviations: ABX: antibacterial therapy, LRTI = lower respiratory tract infection, MR-proADM = mid-regional proadrenomedullin. Size of the impact: red = high-moderate, blue = low, and green = unknown. * = according to previous studies [3,4,5].

Our results could be justified by several overlapping motivations. This heterogeneity reflects the complex origin of CnS. In fact, many reasonable explanations of culture negativity have been hypothesized over time in septic patients [2,6,7,8,9,10,11,12,13,14,15,16,17]. We speculated about the magnitude of their impact on the genesis of CnS in this study, according to the serum concentrations of MR-proADM (Figure 4).

### 4.1. Sepsis Mimickers

Among all the patients with suspected sepsis and negative cultures, 12% were not infected (ni-SIRS), and 12% could not be infected (d-SIRS) [18,20]. The misclassification of ni-SIRS and the quote of non-infected d-SIRS as CnS could justify the dulled concentrations of MR-proADM in CnS [18]. However, this eventuality does not seem applicable to our work, since ni-SIRS and d-SIRS were excluded from this secondary analysis. No studies exist that have clearly identified and excluded both cohorts of patients (especially d-SIRS, which are usually subjected to more levels of adjudication by experts) from the comparison of CnS and CpS.

### 4.2. Insufficient Work Up and Technical Issues

The results of the works conducted to explore the clinical differences between CnS and CpS are heavily influenced by the definition of culture-negative status. Usually, CnS is identified as sepsis with negative blood cultures [2,6,7,8,9,10,11,12,13,14,15,16,17]. In all the aforementioned works but one [15], the rate of collection of biological samples other than blood is not clear. However, it would be advisable to clearly state their contribution to identify the etiology in both CnS and CpS, since it might be inferred that CnS would be the result of an insufficient work-up with respect to CpS if the rate of collection was lower in the former. The impact of this motivation in our study seems very limited since (1) we did not find a significant difference in the rate of sampling of any biological samples between CnS and CpS and (2) an insufficient work-up could not justify the blunted MR-proADM concentrations observed in CnS. Finally, technical issues may have occurred in collecting, storing, transporting, and processing biological samples. From this point of view, our study has a limitation because we did not define the blood culturing methods, or the method used to detect bacteremia in each institution. However, the occurrence of technical problems would not be explainable by the dulled concentrations of serum MR-proADM detected in CnS.

### 4.3. Source of Infection

Several studies demonstrated that

-for certain sources of sepsis (e.g., LRTIs), the frequency of bacteremia was lower than that of other sources (e.g., urinary tract infections) [21]

-if compared with bacteremic patients, non-bacteremic septic patients were associated with lower serum levels of procalcitonin [12,13,15,16,17].

In this study, not only were the median concentrations of procalcitonin significantly lower in patients with LRTIs than in patients with non-LRTIs but also those of sIL2Rα, presepsin, and MR-proADM. Compared to CpS, the same four biomarkers were also proven to be dulled in CnS (Table 1). Since the percentage of LRTIs was significantly higher in CnS than in CpS (*p* < 0.001), we speculated that the blunted levels of procalcitonin, sIL2Rα, presepsin, and MR-proADM found in CnS could be subordinate to the source of infection. However, after correction for the source of infection and the other covariates, MR-proADM was found to be independently correlated with culture-negative status (see Table S2).

### 4.4. Bacterial Virulence and Host Response

CnS could be caused by bacteria exhibiting weaker virulence than those causing CpS. Therefore, compared to CpS, CnS would be associated with a lower bacterial load at the infection site and/or a lower extent/duration of bacteremia. These assumptions could justify both the blunted levels of MR-proADM (epiphenomenon of milder immune system stimulation) and the negativity of cultures. Then, we theorized that the three cultural statuses (BSI, PCOTB, and CnS) could be linked with decreasing virulence and possibly with declining levels of MR-proADM. After correction for covariates, the median concentrations of MR-proADM were confirmed to be significantly reduced starting from BSI, through sepsis with PCOTB, and ending with CnS (Figure 2). This result supports the following hypothetical relations between cultural status, bacterial virulence, and MR-proADM levels: (1) in CnS, the virulence is so weak (lowest MR-proADM levels) that bacteria cannot spread to the bloodstream (negative blood cultures), but also results in it being undetectable in samples of the source of infection; (2) in PCOTB, the extent of virulence (intermediate MR-proADM levels) is such as to determine a low grade/transient bacteremia, making pathogen detection only possible in samples of the source of infection; and (3) in BSI, the high virulence expressed at the infection site by the offending bacteria (highest MR-proADM levels) allows its high grade/sustained spread into the bloodstream, making the pathogen simultaneously culturable in samples of the suspect source of infection and in the blood.

Moreover, bacteremia develops not only when bacteria evade host immune defenses, but also when inherent or acquired immune defects of immune defenses lead to a failure to limit the bacterial spread. Preliminary studies have confirmed that the genetic make-up of the host could play a key role in this sense [22,23,24,25,26,27]. With respect to CpS, we hypothesized that CnS could be associated with a higher efficacy of the immune response in limiting infection. In this case, MR-proADM levels should be inversely connected to the degree of orchestration of the immune response. The correlation between this unique feature of the immune response, genetic background of the host, and MR-proADM levels remains to be defined; however, this hypothesis is supported by the fact that some comorbidities, such as diabetes [28] (associated with a less robust immune response), seem more frequent in the cohort with the highest median concentration of MR-proADM (i.e., BSI; see Table S4). Since MR-proADM concentrations have already been proven to be directly linked to the chances of culturing the offending bacteria we may speculate that: (1) in CnS, none of the biological samples are positive due to the high efficacy of the immune response (lowest MR-proADM) in limiting the bacterial load at the infection site, preventing bacteremia or reducing its extent/duration; (2) in PCOTB, the immune response is able to prevent/clear bacteremia but can’t limit the bacterial load at the infection site (intermediate MR-proADM levels); and (3) in BSI, the immune response is so lowly orchestrated (highest MR-proADM levels) that bacteria is culturable not only at the infection site but also in the blood.

The interplay between bacterial virulence and the efficiency of the immune response could have an impact on the genesis of CnS. However, if the reduced concentrations of MR-proADM were caused by bacteria with weak virulence, a highly orchestrated immune response, or both, CnS should nevertheless be a cohort of patients with a better prognosis than those with CpS. This hypothesis is elicited by the fact that a low concentration of MR-proADM has been clearly associated with a favorable outcome in sepsis studies [29,30]. In our work, we did not observe a significant difference in terms of mortality between CnS and CpS, but CnS exhibits both a lower odds of multi-organ failure and a shorter length of hospital stay than CpS (Table 2).

### 4.5. Previous or Concurrent Antibacterial Therapy?

Concurrent antibacterial therapy could both obscure cultures and justify the blunted MR-proADM levels.

Three [3,4,5] in four studies [31] have demonstrated that the diagnostic yield of blood cultures is made worse by the concurrent administration of antibiotics in septic patients. Even if we lack objective data in this study, we feel that this motivation of culture negativity could significantly impact the genesis of the culture-negative status (94% of patients with CnS received at least one dose of antibacterials during their stay in ED). Furthermore, we registered whether the patients had taken at least one dose of an antibacterial within 30 days from the drawing of all cultures (roughly coinciding with ED admission). Due to the broad time frame between the administration of antibacterial therapy and the collection of cultures (i.e., previous), we can provide information about the impact on the diagnostic yield of cultures for antibacterial therapy concluded at home several days before admission or completed during any recent hospitalizations. After comparing CnS and CpS, we failed to demonstrate that the administration of antimicrobial therapy within 30 days of ED admission significantly reduced the chances of a pathogen being detected either in the blood or in all cultures (*p* = 0.37 and *p* = 0.171, respectively).

### 4.6. Uncommon Bacteria and Non-Bacterial Etiology

Fastidious growth of bacteria, in which prolonged incubation is necessary, and bacteria that cannot be routinely cultured in blood with currently available techniques could contribute to a culture-negative status [2,6,7,8,9,10,11,12,13,14,15,16,17]; for certain sources of infection, their involvement is far from negligible [32]. Studies do not exist that have compared those etiologies with common bacteria in terms of MR-proADM concentrations. In this study, the low percentage of infections caused by these uncommon bacteria does not enable us to provide convincing comparisons.

In our work, MR-proADM levels were lower in those with non-bacterial sepsis compared to that in those with bacterial sepsis (28 and 339 patients, respectively, *p* < 0.001). Therefore, a non-bacterial etiology is undoubtedly implicated in the genesis of CnS. Virtually all non-bacterial pathogens can cause sepsis; however, only some of them are not culturable in blood or in samples of the source of infection. Studies have not assessed the role of MR-proADM in distinguishing bacterial sepsis from viral sepsis or sepsis caused by non-culturable fungi/protozoa. In this study, given the small sample size of non-bacterial etiology, its subgroups were not further compared. However, we feel that viruses can massively impact the genesis of CnS for two reasons. First, after bacteria, viruses represent the most common etiology of community-acquired pneumonia [33], and the lower respiratory tract is the most common source of CnS in our work. Second, the prevalence of a viral etiology for sepsis is below expectations [34] (none of the enrolling centers used multiplex PCR to detect respiratory viruses in patients with LRTIs at ED admission).

The secondary objective of this study was to derive a clinical prediction rule that could predict culture-negative status with reasonable certainty since entrance into the hospital, when the results from microbiological examinations are not available. It would be of paramount importance to support ED physicians in the early identification of patients with CnS. Beyond the benefit of rationalization for the use of cultures, a clinical prediction rule for CnS may have a positive impact on the management of patients with culture-negative status in more ways than one. First, even if it has been clearly demonstrated that prompt administration of antibiotics improves the outcome of sepsis [1], in our study, patients with CnS did not receive antibacterial therapy during their stay in ED more frequently than those with CpS (Table 1). Early identification of patients with CnS may reduce the risk of delay in the administration of this life-saving therapy. Second, CnS represents a cohort of patients at high risk for destabilization for a potentially inappropriate spectrum and/or a suboptimal duration of antibacterial therapy. A clinical prediction rule for CnS could help ED physicians to identify specific trajectories of monitoring for patients with CnS upon their admission to the hospital. Third, there was the absence of microbiological documentation of the exposure for patients with CnS to the risk of adverse events related to an antibacterial therapy that is exceedingly broad in spectrum or too protracted (e.g., *Clostridium difficile* infections and bacterial resistance). In fact, the relation between early antibiotic de-escalation strategies and outcomes have been explored only in CnS. Even if the evidence was low quality, antibacterial stewardship was not associated with worse mortality [35]. The early recognition of patients with CnS could elicit the early de-escalation of antimicrobial therapy, limiting adverse events related to its inappropriate use. Fourth, rapid molecular diagnostic techniques for the identification of the offending pathogen directly from whole blood samples could provide valuable information about the etiology of sepsis with a shorter turnaround time than conventional cultures [36]. Unfortunately, the costs relating to their routine use in both CnS and CpS are high [37]. A screening tool for CnS could support the ED physicians in identifying the patients who mostly need elucidations, since, in contrast to CpS, they lack support in defining the etiology of sepsis by conventional cultures. Given the above premises and the epidemiological data (approximately 50% of sepsis cases are CnS [2,6,7,8,9,10,11,12,13,14,15,16,17]), a clinical prediction rule could halve the costs related to the use of rapid diagnostic technology.

We built a nomogram to predict the pre-test probability of CnS in patients with a definitive diagnosis of sepsis at ED admission (Figure 2a). It’s AUROC was satisfactory (0.84) but far from perfection. The addition of MR-proADM improved the performance of the nomogram based only on the clinical variables (AUROC 0.82, *p* = 0.02). However, even if the nomogram was enriched with MR-proADM, it exhibited low sensitivity, specificity, NPV, and PPV for CnS (0.80, 0.79, 0.71, and 0.86, respectively; Table S3). Further studies are needed to derive and validate a clinical prediction rule that could robustly rule in/out culture-negative status in septic patients when the results of the cultures are not yet available.

Finally, the adoption of the SIRS criteria (Sepsis-2), instead of qSOFA (quick sequential organ failure assessment)/SOFA score (Sepsis-3) [38], to enroll the septic patients may seem a further limit of the study. However, this topic (Sepsis-3 vs. Sepsis-2 for early diagnosis of infection) is still a matter of debate. The SIRS criteria were abandoned due to the high sensitivity and low specificity for infection diagnosis [38]. Up to now, only one work compared the performance of the Sepsis-3 with that of Sepsis-2 in predicting infection diagnosis, which was posed at the end of clinical work-up among patients with suspected infection. Shiraishi et al. retrospectively analyzed the data from a multicenter prospective study [39]. Among the 1045 patients eligible, SIRS outperformed the qSOFA score (the “quick” surrogate of SOFA score^38^) for predicting established infection (AUC 0.65 and 0.58, respectively). Interestingly, SIRS patients include almost all of the qSOFA patients in that paper.

## 5. Conclusions

Out of the six serum biomarkers assayed in this study, only MR-proADM predicted culture-negative status independently from covariates. If compared with PCOTB and BSI, CnS were associated with the lowest median serum concentrations of MR-proADM. Our multivariable nomogram, based on MR-proADM, did not predict culture-negative status with reasonable certainty in patients with definitive diagnosis of sepsis at ED admission.

## Figures and Tables

**Table 1:** Characteristics at baseline.

Characteristics	CnS (n = 449)	CpS (n = 324)	Un *p*	Un *OR*
Male, n (%)	246 (54)	172 (53)	0.639	
Median age (IQR)	82 (73–88)	79 (72–85)	0.156	
Median Charlson comorbidity index (IQR)	3 (1–4)	3 (1–4)	0.736	
Diabetes, n (%)	63 (14)	70 (21)	0.006	0.592 (0.407–0.862)
Chronic heart failure, n (%)	121 (27)	63 (19)	0.016	1.528 (1.082–2.158)
Previous acute myocardial infarction, n (%)	114 (25)	59 (18)	0.019	1.528 (1.074–2.176)
Solid cancer, n (%)	33 (7)	30 (9)	0.339	
Haematologic cancer, n (%)	12 (3)	10 (3)	0.733	
Chronic liver disease, n (%)	33 (7)	28 (9)	0.511	
Chronic pulmonary disease, n (%)	140 (31)	62 (19)	<0.001	1.915 (1.361–2.693)
Chronic kidney disease, n (%)	82 (18)	64 (19)	0.602	
Dementia, n (%)	136 (30)	96 (30)	0.843	
Chronic rheumatologic disease, n (%)	16 (3)	14 (4)	0.591	
AIDS, n (%)	2 (0)	0 (0)	0.999	
Antibacterials within 30 days from ED admission ^, n (%)	129 (29)	108 (33)	0.171	
Patients untreated with antibacterials during their stay in ED *, n (%)	26 (6)	6 (2)	0.009	3.257 (1.325–8.009)
Prosthetic devices, n (%)	52 (11)	74 (23)	<0.001	0.443 (0.300–0.653)
Median body temperature (°C), (IQR)	37.5 (36.6–38.2)	37.8 (36.7–38.2)	0.331	
Meadian mean arterial pressure (mmHg), (IQR)	86 (76–95)	85 (75–93)	<0.001	1.019 (1.009–1.029)
Median heart rate (beats/min), (IQR)	100 (90–110)	104 (78–120)	0.912	
Median respiratory rate (breaths/min), (IQR)	24 (20–28)	24 (21–32)	0.361	
Median Glasgow Coma Scale (IQR)	15 (15–15)	15 (15–15)	0.183	
Median white blood cell count x1000/mm^3^ (IQR)	12.5 (9.0–16.4)	13.4 (9.8–18.5)	0.025	0.806 (0.629–1.001) *
Median hemoglobin (g/L) (IQR)	12.6 (11.0–13.8)	11.6 (10.6–13.0)	<0.001	1.249 (1.159–1.346)
Median platelets count x1000/mm^3^ (IQR)	222 (160–298)	215 (151–300)	0.330	
Median serum urea (mg/dL) (IQR)	43 (30–63)	46 (30–74)	0.033	0.996 (0.993–1.000)
Median creatinine/mg/dL) (IQR)	1.0 (0.8–1.6)	1.1 (0.9–1.7)	0.039	0.873 (0.768–0.993)
Median sodium (mEq/L) (IQR)	137 (134–140)	135 (133–138)	<0.001	1.052 (1.025–1.079)
Median potassium (mEq/L) (IQR)	3.9 (3.5–4.4)	3.9 (3.5–4.4)	0.631	
Median AST (U/L) (IQR)	24 (17–36)	27 (19–59)	0.910	
Median ALT (U/L) (IQR)	17 (11–29)	23 (12–41)	0.456	
Median total bilirubin (mg/dL) (IQR)	0.9 (0.6–1.4)	0.9 (0.7–1.4)	0.152	
Median INR (IQR)	1.1 (1.0–1.3)	1.37 (1.17–3.2)	0.223	
Median fibrinogen (mg/dL) (IQR)	478 (382–637)	495 (378–668)	0.340	
**Biomarkers**				
Median C-reactive protein (mg/dL) (IQR)	81 (31–170)	127 (53–215)	<0.001	0.769 (0.678–0.872)
Median lactate (mg/dL) (IQR)	14 (9–19)	14 (10–21)	0.103	
Median procalcitonin (ng/mL) (IQR)	0.51 (0.16–2.43)	1.12 (0.29–9.51)	<0.001	0.698 (0.619–0.787)
Median sIL2Rα (pg/mL) (IQR)	13367 (9050–16817)	17510 (10635–29856)	<0.001	0.588 (0.481–0.718)
Median sTREM-1 (pg/mL) (IQR)	398 (269–634)	456 (292–742)	0.101	
Median sPLA_2_GIIA (ng/mL) (IQR)	30.8 (24.1–35.5)	32.4 (28.0–36.3)	0.002	0.600 (0.434–0.830)
Median presepsin (pg/mL) (IQR)	525 (321–918)	675 (359–1328)	<0.001	0.645 (0.547–0.762)
Median MR-proADM (nmol/L) (IQR)	1.93 (1.29–3.06)	2.31 (1.45–4.13)	<0.001	0.349 (0.248–0.490)
**Source of infection**				
Single Source, n (%)	413 (92)	266 (82)	<0.001	2.375 (1.501–3.757)
-LRTI, n (%)	339 (82)	71 (27)	<0.001	11.394 (7.974–16.281)
-Non LRTI, n (%)	74 (18)	195 (73)		
Multiple Source, n (%)	36 (8)	58 (18)		
**Microbiological work up**				
At least one biological sample other than blood, n (%)	441 (98)	322 (99)	0.205	
Median SOFA score (IQR)	3 (2–4)	3 (1–5)	0.092	

List of abbreviations: CnS = culture negative sepsis, CpS = culture positive sepsis, Un = unstandardised, OR = Odds ratio, IQR = interquartile, ED = emergency department, AST = aspartate aminotransferase, ALT = alanine aminotransferase, INR = international normalized ratio, sIL2Rα = soluble IL-2 receptor α, sTREM-1 = soluble triggering receptor expressed on myeloid cell-1, sPLA_2_GIIA = soluble phospholipase A_2_ group IIA, MR-proADM = mid-regional proadrenomedullin, LRTI = lower respiratory tract infection, and SOFA = sequential organ failure assessment. ^ at least one dose of antibacterials was administered within 30 days from emergency department admission. * Log scale transformed.

**Table 2:** Outcome at 30 days.

Characteristics	CnS (n = 449)	CpS (n = 324)	*p*
Organ dysfunction *			
Renal	116 (26)	113 (35)	0.007
Cardiovascular	190 (42)	93 (29)	<0.001
-Narrow/broad complex tachycardia	38 (8)	25 (8)	0.790
-Atrial fibrillation	29 (6)	13 (4)	0.092
-Newly detected	10 (2)	6 (2)	0.718
-Acute decompensated heart failure	116 (26)	54 (17)	0.002
-Acute coronary syndrome	68 (15)	25 (8)	0.002
-Shock	26 (6)	16 (5)	0.606
Respiratory	271 (60)	133 (41)	<0.001
Haemostasis	77 (17)	73 (22)	0.062
Haematologic	29 (6)	30 (9)	0.148
Neurologic	122 (27)	114 (35)	0.002
Liver	12 (3)	13 (4)	0.299
Median number of organ	2 (1–3)	2 (1–3)	0.053
-Multiorgan failure	4 (1)	11 (3)	0.013
Median length of hospital stay	9 (7–16)	12 (7–19)	<0.001
Mortality at 30 days	88 (19)	66 (20)	0.791

List of abbreviations: CnS = culture negative sepsis, CpS = culture positive sepsis. * The definition of each organ dysfunction and that of multi-organ failure are available in the Supplementary Materials (PDCS). A significant trend towards a positive linear correlation between serum concentrations of MR-proADM and SOFA score (Spearman rho = 0.478, *p* < 0.001) was observed (Figure S1 available in the Supplementary Materials).

## Data Availability

Individual de-identified participant data are available from the corresponding author on reasonable request.

## Supplementary Materials

The following supporting information can be downloaded at: www.mdpi.com/article/10.3390/biomedicines10020357/s1. References [40,41,42,43,44,45,46] are cited in the supplementary materials. Supplementary information on predefined classification system, flowchart of enrollment in this secondary analysis, etiology of culture-positive bacterial sepsis, independent variables of culture-negative status, performance of the nomogram based on mid-regional proadrenomedullin to predict culture-negative status, and characteristics at baseline and mortality at 30 days of the three cultural statuses.

## Abbreviations

ED: emergency department, CnS: culture-negative sepsis, CpS: culture-positive sepsis, SIRS = systemic inflammatory disease, ni-SIRS: non-infective SIRS, d-SIRS: debatable SIRS, PDCS: predefined classification system, BSI: bloodstream infections, BC=blood cultures, PCOTB: positive cultures other than blood, LRTI: lower respiratory tract infection, non-LRTI: non-lower respiratory tract infection, SOFA: sequential organ failure assessment. AST = aspartate aminotransferase, ALT = alanine aminotransferase, INR = international normalized ratio, sPLA_2_GIIA: soluble phospholipase A_2_ group IIA, sIL2Rα: soluble IL-2 receptor α, sTREM-1: soluble triggering receptor expressed on myeloid cell-1, MR-proADM: mid-regional proadrenomedullin, OR: odds ratios, CIs: confidence intervals, IQR: interquartile, AUROC: area under the receiver-operating characteristic curve, NPV: negative predictive value, PPV: positive predictive value, NLR: negative likelihood ratio, and PLR: positive likelihood ratio.
