# Supplementary material for: The Role of Mid-Regional Proadrenomedullin in the Differential Diagnosis between Culture-Negative and Culture-Positive Sepsis at Emergency Department Admission
Source: Biomedicines. 2022 Feb 1;10(2):357. doi: 10.3390/biomedicines10020357 (PMC8962368; doi:10.3390/biomedicines10020357)
Supplement: Supplementary file 1 [file biomedicines-10-00357-s001.zip › biomedicines-1497822-supplementary.pdf]

Supplementary Materials

Predefined Classification System (PDCS)

The PDCS was based on guidelines established by the main Authorities in the field of infection [40–46] and was approved, before the start of enrolment, after discussion among principal investigators.

Comorbidities

The following comorbidities were collected in this study: prior myocardial infarction (history not solely ECG changes), congestive heart failure, peripheral vascular disease (included aortic aneurysm ≥ 6 cm), chronic dementia (decline in at least 2 brain functions), chronic respiratory disease (include chronic obstructive pulmonary disease [COPD]), structural lung diseases such as bronchiectasis and interstitial lung disease), connective tissue diseases/vasculitis, peptic ulcer, liver disease, diabetes mellitus, chronic renal disease (defined as abnormal basal creatinine), solid cancer (was adjudicated if active at the time of presentation or requiring antineoplastic treatment within the previous five years), hematologic cancer, HIV and related syndromes.

Clinically Documented Infections (C-Infections) and Microbiologically Documented Infections (M-Infections)

C-infections comprised SIRS cases with a clinical history and course suggestive of infection without supporting Gram stain or cultures. M-infections included SIRS cases with microbiological confirmation of infection.

Etiology of Infection

Bacteremia was defined as the presence of viable bacteria in the circulating blood. Occult bacteremia was defined as bacteremia not associated with clear foci of infection. In this study, single blood culture positive for organisms consistent with skin flora (coagulase-negative Staphylococci, Corynebacterium spp, and alpha-hemolytic Streptococci) was considered contaminated. Respiratory samples were considered positive if a bacterial yield in cultures of valid sputum of at least 10^6 CFU/mL or a bacterial yield in cultures of bronchoalveolar lavage (BAL) of at least 10^4 CFU/ml were documented. In this study, Candida spp. isolated from sputum or BAL were considered colonizers unless also present at multiple other sites. Urinary tract infection in men was defined as acute if the symptoms were consistent with the diagnosis and bacteria $\geq 10^4$ CFU/mL were documented on midstream urine specimens. Urinary tract infection in patients with an indwelling catheter was adjudicated by fever >38° C, pain above the pubic bone or in the flank, in addition to one of the following: urine culture with $\geq 10^5$ CFU/mL regardless of the results of the urinalysis, urine culture with $\geq 10^3$ CFU/mL and evidence of pyuria. Specimens were sent for culture only when skin, soft tissue, and bone infections were suspected. Sepsis was considered microbiologically documented only in cases of positive microbiological results derived from deep tissue by biopsy or curettage; otherwise, sepsis was considered clinically documented.

Source of Infection

When clinical work-up revealed one source of infection, the case was classified as a single source of infection; if at least two unrelated sources of infection were identified, the case was classified as infection with multiple sources. Lower respiratory tract (LRT), urinary tract, skin, soft tissues and bone, and abdominal infections were defined according to guidelines of the Infectious Diseases Society of America [40–46]. We used the acronym LRTI to identify patients with a single source of infection localized in LRT; the other single sources of infection were grouped as non-LRTI.

Severity of Infection, and Definitions of Organ Dysfunction

Uncomplicated infection was a SIRS with a definite infective etiology and absence of organ dysfunction. Sepsis was defined as infection associated with signs of hypoperfusion (such as mottled skin, capillary refill ≥ 3 secs, urine output <0.5 mL/kg for at least one hour, or needing dialysis, blood lactate > 18 mg/dL) or organ dysfunction. Organ dysfunction was defined as present when any of the following criteria were met: SaO2 < 90%* or PaO_2/FiO_2 < 300* or ARDS defined as PaO_2/FiO_2 <300 and bilateral diffuse opacities, not suggestive of pleural effusion, atelectasis or nodules on bilateral chest X-ray (respiratory dysfunction), increase in serum creatinine of 0.3 mg/dL or ≥ to 1.5 fold from baseline within 48 hours (renal dysfunction), platelets count < 100000/mm^3 ** (hematologic dysfunction), apTT > 60 sec** or INR> 1.2** or disseminated intravascular coagulation (haemostasis dysfunction), total bilirubin > 4 mg/dL* or INR > 1.5** (liver dysfunction), any form of mental change, inattention, and disorganized thinking indicative of encephalopathy or delirium or any degree of Glasgow Coma Scale worsening (neurologic dysfunction), cardiac arrhythmias*** as high rate atrial fibrillation, high rate narrow complex, ventricular tachycardia, acute coronary syndromes, and acute decompensated heart failure*** (cardiovascular dysfunction). High-rate atrial fibrillation or narrow complex tachycardia referred to patients who were clinically or hemodynamically unstable (i.e. myocardial ischemia, pulmonary edema, or hypotension). Septic shock was defined as sepsis plus one or both of the following conditions: mean systemic arterial pressure <60 mmHg (or <80 mmHg compared to usual pressure rates) despite adequate fluid-resuscitation strategy, and need for dopamine, norepinephrine, or epinephrine, despite the administration of fluids required to maintain mean arterial pressure >60 mmHg (or >80 mmHg if patient has hypertension).

*not known to be chronic
**not known to be chronic or due to medications
***during the concurrent hospital stay for sepsis, but not primary diagnosis

Multiorgan dysfunction was defined as sepsis with least three of the organ dysfunctions mentioned above.

Statistical Process to Generate the Nomogram

The first step was to estimate a multivariable logistic regression model for predicting the risk of culture negative sepsis; univariate odds ratios and then multivariate logistic regression analysis was used for identifying variables associated with the risk of culture negative sepsis, as described in the Statistical analysis section of the paper. This model provided the equation used to generate a Nomogram. The Nomogram calculates the individual probability of culture negative sepsis based on covariates values and regression weights. In brief: taking into account the regression Beta estimates of the model and the range of values that each predictor has in the population, it is possible to compute the "absolute maximum beta values" multiplying the betas by the range of predictors; in this way, the predictor that has the biggest impact in the model is identified, and a score of 0 is assigned to its lowest value and a score of 100 to its highest value. Points to the other predictor's values are assigned based on linear interpolation. Then, for the other predictors, ranked in order of decreasing impact, maximum points are assigned on the basis of a proportion of their "absolute maximum beta value" with respect to the one of the first predictor. The minimum point of zero is always assigned to the lowest value of each predictor. For a hypothetical patient, points from each predictor are summed to obtain a total score. When the total score is available, to project it on the probability scale the "points per unit of linear predictor" and the "linear predictor units per point" are computed. Using the inverse of the link function of the logistic model, the probability of infection is finally derived. This has been done through the function "nomogram" in the R package "rms", using the software R. Specifically in our case:

Linear predictor= Absence of Diabetes (Yes)*0.8988+Hemoglobin*0.1487+ LRTI *2.5512-Log(MR-proADM)*0.936
Costant= -2.2597
Example: Reading from the graph
Absence of Diabetes Yes= 24 points
Hemoglobin=8= 20 points
LRTI yes= 68 points
Log(MR-proADM)=1.5= 62 points
Total points= 174 -> around 70% culture negative sepsis
Calculating from the equation:
Linear Predictor= 1*0.8988+8*0.1487+ 1*2.5512-1.5*0.936=3.2356
predprob = exp(-2.2597+3.2356)/(1+exp(-2.2597+3.2356))
predprob 0.73 (73% risk of infection)

References

40. Dellinger, R.P.; Levy, M.M.; Rhodes, A., et al. Surviving Sepsis Campaign: international guidelines for the management of severe sepsis and septic shock, 2012. *Intensiv. Care Med*. **2013**, *39*, 165–228.
41. Baron, E.J.; Miller, J.M.; Weinstein, M.P., et al. Executive summary: a guide to utilization of the microbiology laboratory for diagnosis of infectious diseases: 2013 recommendations by the Infectious Diseases Society of America (IDSA) and the American Society for Microbiology (ASM). *Clin. Infect. Dis.* **2013**, *57*, 485–8.
42. Woodhead, M.; Blasi, F.; Ewing, S. et al. Guidelines for the management of adult lower respiratory tract infections. *Clin. Microbiol. Infect.* **2011**, *17*, (Suppl 6), E1–59.
43. Mandell, L.A.; Wunderink, R.G.; Anzueto A, et al. Infectious Diseases Society of America/American Thoracic Society consensus guidelines on the management of community-acquired pneumonia in adults. *Clin. Infect. Dis.* **2007**, *44*, (Suppl 2), S27–72.
44. Gupta, K.; Hooton, T.M.; Naber KG, et al. International clinical practice guidelines for the treatment of acute uncomplicated cystitis and pyelonephritis in women: A 2010 update by the Infectious Diseases Society of America and the European Society for Microbiology and Infectious Diseases. *Clin. Infect. Dis.* **2011**, *52*, e103–20.
45. Stevens, D.L.; Bisno, A.l.; Chambers, H.F., et al. Practice guidelines for the diagnosis and management of skin and soft tissue infections: 2014 update by the infectious diseases society of America. *Clin. Infect. Dis.* **2014**, *59*, 147–59.
46. Solomkin, J.S.; Mazuski, J.E.; Bradley, J.S. et al. Diagnosis and management of complicated intra-abdominal infection in adults and children: guidelines by the Surgical Infection Society and the Infectious Diseases Society of America. *Surg. Infect.* (Larchmt) **2010**, *11*, 79–109.

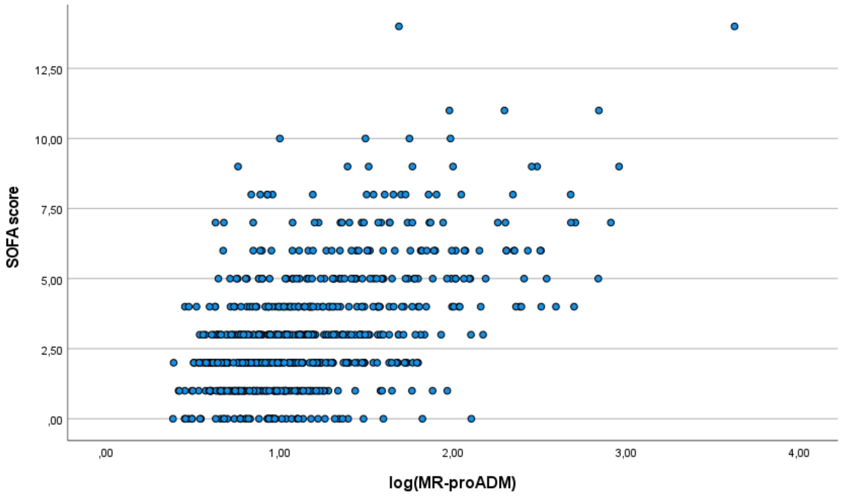

Figure S1. Correlation between serum concentrations of mid-regional proadrenomedullin (MR-proADM) and Sequential Organ Failure Assessment (SOFA) score. Being the SOFA score a calculated variable that can assume only positive integers, values points were "clustered" according to the one-unit SOFA score change.

Table S1. Etiology of culture-positive bacterial sepsis.

PATHOGENS	*n*
**Gram positive**	**160**
Coagulase-negative stafilococci	10
Stafilococcus aureus	50
Streptococcus pneumoniae	10
Viridans streptococci	16
Streptococcus gallolyticus	5
Enterococcus faecalis	27
Enterococcus faecium	8
Streptococcus agalactiae	5
Streptococcus pyogenes	1
Clostridium difficile	20
Other Gram positive	8
**Gram negative**	**264**
Escherichia coli	126
Pseudomonas aeruginosa	33
Non-aeruginosa Pseudomonas	1
Haemophilus influenzae	6
Haemophilus parainfluenzae	1
Stenotrophomonas maltophila	4
Klebsiella oxytoca	4
Klebsiella pneumoniae	25
Proteus mirabilis	24
Proteus vulgaris	1
Citrobacter freundii	4
Citrobacter koseri	2
Morganella morganiii	5
Serratia marcescens	3
Providencia stuarti	1
Enterobacter cloacae	8
Acinetobacter baumanii	5
Bacteroides fragilis	1
Bacteroides stercoris	1
Campylobacter jejunii	2
Other Gram negative	7
Total	**424**

Table S2. Independent variables of culture-negative status.

Variables	B	SE	Wald	Adjusted p	Adjusted Odds Ratio
Absence of diabetes	0.899	0.294	9.324	0.002	2.457
Hemoglobin	0.149	0.055	7.431	0.006	1.160
Lower respiratory tract	2.551	0.224	129.665	<0.001	12.822
Mid-regional proadrenomedullin	-0.936	0.230	16.545	<0.001	0.392

List of abbreviations B=Beta coefficient and SE=standard deviation.

Table S3. Performance of the nomogram, based on mid-regional proadrenomedullin, to predict culture-negative status.

Sensitivity	Specificity	NPV	PPV	NLR	PLR
0.80<br>(0.75-0.84)	0.79<br>(0.73-0.84)	0.71<br>(0.65-0.78)	0.86<br>(0.81-0.89)	0.25<br>(0.20-0.32)	3.83<br>(2.93-5)

()=95% confidence interval. List of abbreviations: NPV=negative predictive value, PPV=positive predictive value, NLR= negative likelihood ratio, and PLR=positive likelihood ratio.

Table S4. Characteristics at baseline and mortality at 30 days.

Characteristics	CnS (n= 449)	PCOTB (n= 171)	BSI (n=153)	*p**
**Demographics**				
Male, n%	246 (54)	89 (52)	83 (54)	0.540;0.692;0.908
Median age (IQR)	82 (73-88)	79 (73-85)	78 (70-85)	0.044;0.358;0.005
**Comorbidities**				
Median Charlson Index (IQR)	3 (1-4)	3 (1-4)	3 (1-5)	0.5151;0.783;0.820
Diabetes , n%	63 (14)	32 (19)	38 (25)	0.148;0.181;0.002
Chronic heart failure, n%	121 (27)	33 (19)	30 (20)	0.047;0.944;0.070
Previous acute myocardial infarction,	114 (25)	33 (19)	26 (17)	0.108;0.592;0.034
n%	33 (7)	11 (6)	19 (12)	0.691;0.064;0.054
Solid cancer, n%	12 (3)	4 (2)	6 (4)	0.815;0.411;0.433
Haematologic cancer, n%	33 (7)	7 (4)	21 (14)	0.140;0.02;0.017
Chronic liver disease, n%	140 (31)	38 (22)	24 (16)	0.026;0.135; <0.001
Chronic pulmonary disease, n%	82 (18)	29 (17)	35 (23)	0.697;0.182;0.213
Chronic kidney disease, n%	136 (30)	60 (35)	36 (23)	0.258;0.023;0.110
Dementia, n%	16 (3)	10 (6)	4 (3)	0.207;0.153;0.572
Chronic rheumatologic disease, n%	2 (0)	0 (0)	0 (0)	0.382 ;1.000, 0.408
AIDS, n%				
Prosthetic devices, n%	52 (12)	33 (19)	41 (27)	0.013;0.108; <0.001
Antibacterials within 30 days from ED admission^, n%	129 (29)	66 (39)	42 (27)	0.001;0.034;0.762
**Objective examination**				
Median body temperature (°C) (IQR)	37.5 (36.6-38.2)	37.5 (36.3-38)	38 (37.1-38.5)	0.172; <0.001;0.003
Median mean arterial pressure	86 (76-95)	86 (76-93)	83 (75-93)	0.076;0.145; <0.001
(mmHg) (IQR)	100 (90-110)	100 (90-110)	100 (90-110)	0.940;0.970;0.993
Median heart rate (beats/min) (IQR)	24 (20-28)	24 (20-26)	24 (20-26)	0.651;0.957;0.649
Median respiratory rate (breaths/min)	15 (15-15)	15 (15-15)	15 (15-15)	0.382;0.855;0.505
(IQR)				
Median Glasgow Coma Scale (IQR)				
**Laboratory**				
Median white blood cell count	12.5 (9.0-16.4)	14 (10.5-18)	12.5 (8.6-18.1)	0.001;0.034;0.684
x1000/mm^3 (IQR)	12.6 (11.0-13.8)	11.8 (10.2-13.1)	11.5 (10.2-12.5)	<0.001;0.099;<0.00
Median hemoglobin (g/L) (IQR)	222 (160-298)	243 (173-322)	178 (129-261)	1
Median platelets count	43 (30-63)	46 (30-72)	46 (33-75)	0.045;

x1000/mm^3(IQR)	1.0 (0.8-1.6)	1.0 (0.8-1.8)	1.2 (0,9-1.9)	<0.001;<0.001
Median serum urea (mg/dl) (IQR)	137 (134-140)	136 (133-139)	134 (132-137)	0.251:0.661;0.087
Median creatinine /mg/dl) (IQR)	3.9 (3.5-4.4)	3.9 (3.4-4.5)	3.9 (3.6-4,4)	0.252;0.120;0.002
Median sodium (mEq/L) (IQR)	24 (17-36)	22 (16-44)	36 (21-71)	0.084;0.002; <0.001
Median potassium (mEq/L) (IQR)	17 (11-29)	17 (11-32)	24 (16-48)	0.746;0.802;0.979
Median AST (U/L) (IQR)	0.9 (0.6-1.4)	0.9 (0.7-1.2)	1.2 (0.7-2.2)	0.744;
Median ALT (U/L) (IQR)	1.1 (1.0-1.3)	1.1 (1.2-1.9)	1.2 (1.1-1.3)	<0.001;<0.001
Median total bilirubin (mg/dl) (IQR)	478 (382-637)	506 (396-650)	480 (357-690)	0.672;
Median INR (IQR)				<0.001;<0.001
Median fibrinogen (mg/dl) (IQR)				0.431;0.002;0.003
				0.283;0.251;0.014
				0.384;0.867;0.609
**Biomarkers**				
Median C-reactive protein (mg/dl)	81 (31-170)	127 (47-208)	127 (56-231)	0.004;0.202; <0.001
(IQR)	14 (9-19)	12 (9-18)	13 (9-19)	0.206;0.001;0.002
Median lactate (mg/dl) (IQR)	0.51 (0.16-2.43)	0.5 (0.2-2.9)	4.34 (0.63-24.23)	0.332;
Median procalcitonin (ng/ml) (IQR)	13367 (9050-16817)	14605	21443	<0.001;<0.001
Median sIL2Rα (pg/ml) (IQR)	398 (269-634)	(9479-24618)	(11990-40174)	0.072;
Median sTREM-1 (pg/ml) (IQR)	30.8 (24.1-35.5)	472 (309-733)	450 (277-747)	<0.001;<0.001
Median sPLA$_2$GIIA (ng/ml) (IQR)	525 (321-918)	31.6 (26.5-36.1)	33.5 (29.4-36.9)	0.018;0.621;0.111
Median presepsin (pg/ml) (IQR)	1.93 (1.29-3.06)	503 (322-1004)	965 (436-1999)	0.197;0.012; <0.001
Median MR-proADM (nmol/L) (IQR)		2.02 (1.34-3.25)	2.85 (1.79-4.76)	0.165;
				<0.001;<0.001
				0.01; <0.001;
				<0.001
**Source of infection**				
Single Source, n%	413 (92)	134 (78)	132 (86)	<0.001;0.081;0.053
-LRTI, n%	339 (82)	39 (29)	34 (26)	<0.001;0.583<0.00
-Non LRTI, n%	74 (18)	95 (71)	98 (74)	1
Multiple Source, n%	36 (8)	37 (22)	21 (14)	
Median SOFA(IQR)	3 (2-4)	2 (1-4)	3 (2-5)	0.246;
				<0.001;<0.001
Mortality at 30 days, n%	88 (19)	34 (20)	32 (21)	1,00;0.890; 0.726

List of abbreviations: CnS=culture-negative sepsis, PCOTB=positive cultures other than blood, BSI=bloodstream infections, IQR=interquartile, ED=emergency department, AST=aspartate aminotransferase, ALT=alanine aminotransferase, INR=international normalized ratio, sIL2Rα=soluble IL-2 receptor α, sTREM-1=soluble triggering receptor expressed on myeloid cell-1, sPLA$_2$GIIA=soluble phospholipase A$_2$ group IIA, MR-proADM=mid-regional proadrenomedullin, LRTI=lower respiratory tract infection, and SOFA=sequential organ failure assessment. ^at least one dose of antibacterials was administered within 30 days from emergency department admission. *p= culture negative vs PCOTB, PCOTB vs BSI, and culture negative vs BSI.
